# Supplementary material for: Electrochemotherapy vs radiotherapy in the treatment of primary cutaneous malignancies or cutaneous metastases from primary solid organ malignancies: A systematic review and narrative synthesis
Source: PLoS One. 2023 Jul 13;18(7):e0288251. doi: 10.1371/journal.pone.0288251 (PMC10343145; doi:10.1371/journal.pone.0288251)
Supplement: S1 Protocol — (PDF) [file pone.0288251.s002.pdf]

Electrochemotherapy vs radiotherapy in the treatment of primary cutaneous malignancies or cutaneous metastases from primary solid organ malignancies: A systematic review and meta-analysis

Review methods were amended after registration. Please see the revision notes and previous versions for detail.

Citation

Angus McMillan, Luke McElroy, Lorcan O'Toole, Paolo Matteucci, Joshua Totty. Electrochemotherapy vs radiotherapy in the treatment of primary cutaneous malignancies or cutaneous metastases from primary solid organ malignancies: A systematic review and meta-analysis. PROSPERO 2021 CRD42021285415 Available from: https://www.crd.york.ac.uk/prospero/display_record.php?ID=CRD42021285415

Review question

Is there any evidence that electrochemotherapy is of equal or greater benefit than radiotherapy in the management of locally advanced or metastatic cutaneous malignancies

Searches [1 change]

MEDLINE, Embase and CINAHL will be searched from the time period from database inception to 28 December 2021. Additionally, searches of trial registries CENTRAL and ClinicalTrials.gov will be undertaken to identify relevant trials. Supplementary searches of the grey literature via Web of Science, Scopus and Zetoc will also be undertaken.

Types of study to be included [1 change]

Randomised control trials, cohort-studies, case control studies and case series will all be eligible for inclusion in the review. Case series will not be eligible for inclusion in any meta-analysis. There will be no limitations made based upon patient selection criteria or study size. Letters, opinion pieces, literature reviews, and case reports will all be excluded.

Condition or domain being studied [1 change]

Primary cutaneous malignancies or cutaneous metastases from primary solid organ malignancies

Participants/population [1 change]

Participants will be patients diagnosed with either primary cutaneous malignancies or cutaneous metastases from other primary solid organ malignancies. There will be no restriction to inclusion based patient demographics, clinical setting, tumour types, or anatomical location of neoplasm. To reduce heterogeneity, any data reported on the treatment of lymph node metastases or metastases from haematological malignancies will be excluded. If this data is inseparable from data reported on primary cutaneous malignancies or cutaneous metastases from other primary solid organ malignancies then the study will be excluded from the review.

Intervention(s), exposure(s) [1 change]

All publications reporting on the use of electrochemotherapy for the treatment of primary cutaneous malignancies or cutaneous metastases from solid organ malignancies will be included. In vitro and animal studies will be excluded. Any studies reporting on multiple treatment modalities will only be included if data on use of electrochemotherapy is distinguishable and additional treatment modalities are appropriately matched with the comparator group.

Comparator(s)/control

The comparator for this study is radiotherapy, delivered as a monotherapy, either with palliative or curative intent.

Main outcome(s) [1 change]

The primary outcome will be tumour volume response according to the Response Evaluation Criteria in Solid Tumors (RECIST) criteria:

- Complete response: disappearance of tumour at 4 weeks

- Partial response: tumour volume reduction by 30% or more at 4 weeks

- Stable disease: neither partial response or progressive disease criteria fulfilled

- Progressive disease: tumour volume increase by 20% or more with no previous complete or partial response or stable disease documented before increased disease

- Objective response: complete response plus partial response

Studies reporting their tumour volume response outcome according to the WHO criteria will have their results for partial response and progressive disease considered adequately similar to the same tumour response categories reported by those using RECIST criteria.

A complete or objective response will be recorded as a successful primary outcome.

Measures of effect

Dichotomous data will be analysed as odds ratios (OR) with a corresponding 95% confidence interval (95% CI) and continuous data as either mean difference (MD) or standardised mean difference (SMD)

Additional outcome(s) [1 change]

Any secondary outcomes reported by included studies will also be examined including but not limited to progression-free survival, patient reported quality of life, and amenability to future successful surgical resection. Any outcomes related to safety will also be collected included rates of pain, side-effects and adverse events.

Measures of effect

Dichotomous data will be analysed as odds ratios (OR) with a corresponding 95% confidence interval (95% CI) and continuous data as either mean difference (MD) or standardised mean difference (SMD)

Data extraction (selection and coding) [1 change]

All data collection and analysis will be undertaken in accordance with the Cochrane Handbook for Systematic Reviews of Interventions. Two authors (AM and LM) will individually extract the data and record it in a pre-designed electronic form. The two authors will compare collected data and if a consensus cannot be reached a third author (JT) will resolve any disagreement between authors.

The following data will be collected for comparison:

1. Study characteristics, funding source, patient demographics, response evaluation time, recruitment/sampling procedures and tumour volume response evaluation method

2. Tumour anatomy, number, size and histotype

3. Electrochemotherapy agent, route and operating procedure technique

4. Radiotherapy technique and characteristics

5. The primary outcome which is tumour volume response reported by the study and any secondary outcomes reported by the study such as patient reported outcome measures, pain, toxicity/adverse events and progression-free survival

If unable to directly extract any of these data from a study, the authors of the study will be contacted by email. If there is no response the study will be excluded from analysis and this outcome will be recorded on the PRISMA flowchart.

Risk of bias (quality) assessment [1 change]

For each study included in the review which incorporates randomisation of participants, a risk of bias assessment will be undertaken using the Cochrane Collaboration's tool for assessing risk of bias in randomised trials. For each study included in the review which does not randomise participants, a risk of bias assessment will be undertaken using the ROBINS-I tool. These tools will be used to stratify studies into those at low, moderate, and high risk of bias. The quality of evidence for each study outcome will be appraised using the Grading of Recommendations, Assessment, Development and Evaluation (GRADE) approach.

A funnel plot will be used to assess the the included studies for publication bias, with a corresponding formal statistical test (Egger's test) to assess for asymmetry that may indicate publication bias.

Strategy for data synthesis [1 change]

Where appropriate and feasible, we will use random effects meta-analysis (RMA) to synthesise results using an appropriate statistical software. An appropriate estimator of variance will be used based upon the number of studies identified and the sample size of each study. A Knapp-Hartung adjustment will be used to account for uncertainty in between-study heterogeneity. Odds ratios will be presented as the meta-analysis statistic and forest plots will be used for graphical representation.

Statistical heterogeneity of compared studies will be calculated and presented using the I^2 statistic. Interpretation of this will be according to the following guideline:

- 0% to 40%: heterogeneity might not be important

- 30% to 60%: may represent moderate heterogeneity

- 50% to 90%: may represent substantial heterogeneity

- 75% to 100%: considerable heterogeneity

If we find substantial heterogeneity, we will report this finding and discuss possible reasons for this in our final report. We will also look to explore potential sources of heterogeneity through pre-planned subgroup analysis.

Should a formal meta-analysis not be feasible or possible due to concerns over a lack of data, or due to clinical heterogeneity of data, results will be presented in a narrative synthesis.

Analysis of subgroups or subsets [1 change]

- Tumour histotype (eg cutaneous melanoma vs cutaneous SCC vs metastatic deposit of other/unknown primary)

- Treatment regime (eg studies following European Standard Operating Procedures on Electrochemotherapy (9) vs non-standard regime for ECT)

- Small (defined as ≤3 cm) and large (defined as >3 cm) tumours

- Therapy delivered with curative vs palliative intent

Contact details for further information

Angus McMillan

angusmcmillan@doctors.net.uk

Organisational affiliation of the review

Hull University Teaching Hospitals NHS Trust

Review team members and their organisational affiliations [1 change]

Dr Angus McMillan. York and Scarborough Teaching Hospitals Foundation Trust

Dr Luke McElroy. Forth Valley Royal Hospital

Dr Lorcan O’Toole. Hull University Teaching Hospitals NHS Trust

Mr Paolo Matteucci. Hull University Teaching Hospitals NHS Trust

Mr Joshua Totty. Centre for Clinical Sciences, Hull York Medical School; Hull University Teaching Hospitals NHS Trust

Type and method of review [1 change]

Intervention, Meta-analysis, Narrative synthesis, Systematic review

Anticipated or actual start date

21 November 2021

Anticipated completion date

01 April 2022

Funding sources/sponsors [1 change]

No direct funding has been secured for the development of this protocol or subsequent review. Joshua Totty is a Clinical Lecturer (CL-2020-03-001) funded by Health Education England (HEE) / National Institute for Health Research (NIHR). The views expressed in this publication are those of the author(s) and not necessarily those of the NIHR, NHS or the UK Department of Health and Social Care

Conflicts of interest

Language

English

Country

England

Stage of review [1 change]

Review Completed not published

Subject index terms status

Subject indexing assigned by CRD

Subject index terms

Electrochemotherapy; Humans; Neoplasms; Radiation Oncology

Date of registration in PROSPERO

17 November 2021

Date of first submission

16 November 2021

Stage of review at time of this submission [2 changes]

**Stage**	**Started**	**Completed**
Preliminary searches	Yes	Yes
Piloting of the study selection process	Yes	Yes
Formal screening of search results against eligibility criteria	Yes	Yes
Data extraction	Yes	Yes
Risk of bias (quality) assessment	Yes	Yes
Data analysis	Yes	Yes

Revision note

Awaiting publication, review complete.

The record owner confirms that the information they have supplied for this submission is accurate and complete and they understand that deliberate provision of inaccurate information or omission of data may be construed as scientific misconduct.

The record owner confirms that they will update the status of the review when it is completed and will add publication details in due course.

Versions

17 November 2021

07 January 2022

05 April 2022

18 November 2022
