# Supplementary material for: Electrochemotherapy vs radiotherapy in the treatment of primary cutaneous malignancies or cutaneous metastases from primary solid organ malignancies: A systematic review and narrative synthesis
Source: PLoS One. 2023 Jul 13;18(7):e0288251. doi: 10.1371/journal.pone.0288251 (PMC10343145; doi:10.1371/journal.pone.0288251)
Supplement: S1 Search strategy — (PDF) [file pone.0288251.s003.pdf]

S3 – Search Strategy

Table S3 outlines the MeSH and free-text terms used in the search strategy applied to the Healthcare Database Advanced Search Tool which was used to search MEDLINE, Embase and CINAHL databases from the time period from database inception to 28 December 2021.

The CENTRAL registry for reviews and for trials was searched using the same combination of MeSH and free-text terms. The ClinicalTrials.gov registry was searched using the term 'electrochemotherapy'.

The free-text terms from the strategy outlined in Table S3 was used to search Web of Science and SCOPUS. The Web of Science results were limited to proceedings papers, meeting abstracts, book chapters, editorial materials, early access and letters. The SCOPUS results were limited to conference papers, letters, book chapters, editorials, short surveys and conference reviews.

A general search was undertaken on Zetoc using the search 'electrochemotherapy AND radiotherapy'.

Table S3. The MeSH and free-text terms used in the database search strategy.

	Concept 1	AND	Concept 2	AND	Concept 3
**MeSH Term**					
**OR**	skin neoplasms		electrochemotherapy		radiotherapy
**OR**	neoplasms, squamous cell		electroporation		
**OR**	neoplasms, basal cell				
**OR**	carcinoma, squamous cell				
**OR**	carcinoma, basal cell				
**OR**	carcinoma, Merkel cell				
**OR**	melanoma				
**OR**	sarcoma				
**OR**	sarcoma, Kaposi				
**Free Text Key Words**					
**OR**	cutaneous ADJ (carcinoma* OR Neoplasm* OR malignanc* OR metastas* OR cancer*)		electrochemotherap*		radiotherap*
**OR**	skin ADJ (carcinoma* OR Neoplasm* OR malignanc* OR metastas* OR cancer*)		electroporat*		radiation ADJ (therap* OR treatment*)
**OR**	"Squamous Cell" ADJ (carcinoma* OR Neoplasm* OR malignanc* OR metastas* OR cancer*)		ECT		electron ADJ (therap* OR treatment* OR beam OR radiat*)
**OR**	"Basal Cell" ADJ (carcinoma* OR Neoplasm* OR malignanc* OR metastas* OR cancer*)				x?ray ADJ (therap* OR treatment* OR beam OR radiat*)
**OR**	"Merkel Cell" ADJ (carcinoma* OR Neoplasm* OR malignanc* OR metastas* OR cancer*)				kilovoltage ADJ (therap* OR treatment* OR beam OR radiat*)
**OR**	melanoma*				kv ADJ (therap* OR treatment* OR beam OR radiat*)
**OR**	metastas*s				
**OR**	sarcoma*				
**OR**	"Kaposi* sarcoma*"				
