# Supplementary material for: Electrochemotherapy vs radiotherapy in the treatment of primary cutaneous malignancies or cutaneous metastases from primary solid organ malignancies: A systematic review and narrative synthesis
Source: PLoS One. 2023 Jul 13;18(7):e0288251. doi: 10.1371/journal.pone.0288251 (PMC10343145; doi:10.1371/journal.pone.0288251)
Supplement: Attachment — Submitted filename: Response to Reviewers.docx [file pone.0288251.s004.docx]

**Reviewer #1**

Comment 1 – I have read with great interest this systematic review about the role of electrochemotherapy for skin malignancies, in comparison with radiotherapy. The paper is well written and data are properly collected and presented. Correctly, the authors highlight the paucity of literature about the potential role of electrochemotherapy with very few experiences available. Nonetheless, I think that this article may help clinicians in the knowledge of this therapeutic option.

*We thank the reviewer for their kind comments.*

Comment 2 – I only suggest to mention, in the Introduction, among the radiotherapy experiences for skin malignancies:

-doi: 10.1007/s00403-021-02268-1.

-doi: 10.4081/rt.2017.6942

*We thank the reviewer for their suggestion. We found the articles they suggested interesting and relevant to the experience of radiotherapy. We have updated the manuscript to include these articles (paragraph 1, page 4).*

**Reviewer #2**

Comment 1 – In Table 1 it would be useful for treating clinicians to know if there were any reported prior treatments to electrochemotherapy/RT to understand if these have a role in resistant or progressive disease. It would also be helpful to know how responses were measured in these studies (RECIST, radiological, clinically etc).

*We thank the reviewer for their suggestion. We have updated Table 1 (page 10) to include more comprehensive details of the two included studies including previous therapies and the method of tumour response assessment.*

Comment 2 – The study by Amaral et al. reported on PFS outcomes with electrochemotherapy vs RT (line 253, page 13) - this should be included in the summary results table on page 12 as PFS is a clinically relevant outcome when deciding between treatment options

*We thank the reviewer for their suggestion. We have updated Table 1 (page 10) to include more comprehensive details of the two included studies including the progression free survival outcome reported by Amaral et al.*

Comment 3 – Similarly, cohort details of the 2 provided studies should be provided in the summary table - as the fact that Robertson et al.'s study was limited to patients with epidermolysis bullosa was not mentioned until much later (line 258, page 13). This is relevant as these results may not be as readily generalisable to clinicians treating cutaneous SCC beyond this rare subgroup

*We thank the reviewer for their suggestion. We have updated Table 1 (page 10) to include more comprehensive details of the two included studies including characteristics of the study populations.*

Comment 4 – As acknowledged in the discussion, page 15 paragraph 1, the two studies are far too heterogenous both in design and in outcome reporting to allow for meaningful conclusions of electrochemotherapy vs radiotherapy to be drawn. This is a major issue with the review paper overall as it is not informative enough to guide practice, and this should be emphasised more clearly in the Abstract and conclusions.

*We thank the reviewer for their suggestion. We have updated the conclusions section of the abstract (paragraph 2, page 3) and in the review conclusion (paragraph 2, page 18) to highlight this issue more clearly.*

Comment 5 – Paragraph 2, page 15 is a repetition from the Results, and does not need to be highlighted again

*We thank the reviewer for their suggestion. We have removed this paragraph and updated the "studies excluded and reasons for exclusion” section to clarify we contacted the authors to obtain extractable data (paragraph 2, page 11). We have also included a short paragraph in the discussion to succinctly emphasise that though there is data on these therapies in the literature, we encountered significant challenges in extracting comparative data in this review (paragraph 2, page 15 to paragraph 1, page 16).*

Comment 6 – Paragraph 3, page 15 is a description of studies identified in the literature search and should be moved to the Methods

*We thank the reviewer for their comments. It is our understanding that conventionally, the outcome of article screening, including the exclusion of studies and the reason for their exclusion, is a result of the study, and should be described within the results section of the manuscript. We have therefore moved this paragraph in the results section (paragraph 5, page 11, to paragraph 1, page 12). If the editorial decision is that this is the wrong place, we will gladly change it. We have also included a short paragraph in the discussion to succinctly emphasise that though there is data on these therapies in the literature, we encountered significant challenges in extracting comparative data in this review (paragraph 2, page 15 to paragraph 1, page 16).*

Comment 7 – There needs to be more text in the Discussion describing the role of electrochemotherapy or radiotherapy in the current treatment landscape of cutaneous metastases/solid tumours - these are not the only treatment options for patients and by not mentioning alternatives (isolated limb perfusion/infusion, systemic medications including checkpoint inhibitors for cSCC/melanoma or HH inhibitors for BCC, intralesional therapies such as T-VEC or IL-2 etc) the manuscript implies clinicians are limited to these two options

*We thank the reviewer for their suggestion. Though the primary aim of this review was to compare electrochemotherapy with radiotherapy, we have included further detail in the discussion to highlighted the existence of other available therapies (paragraph 2, page 17).*

Comment 8 – The practical benefits for patients in comparison to these alternatives should be discussed in more detail - e.g. reduced time/financial toxicities, reduced systemic side effects etc

*We thank the reviewer for their suggestion. Though the primary aim of this review was to compare electrochemotherapy with radiotherapy we have included additional detail within the discussion to examine some of the advantages and disadvantages of electrochemotherpay and radiotherapy compared with other treatment modalities (paragraph 2, page 17).*

Comment 9 – All in all, I do not think the narrow range of results and conclusions that can be drawn from this study justify publication as such as lengthy article. It would be more suitable to shorten it to a Letter or Correspondence format; or to expand upon the search strategy to include single-arm studies of either electrochemotherapy or radiotherapy in cutaneous metastases to provide a more meaningful indirect comparison (given that the two provided studies are both non-randomised and heterogenous themselves).

*We thank the reviewer for their comment. We have taken great care to outline the paucity of evidence on this topic, and to report this in a succinct fashion. PLOS has no submission type for letter/short correspondence, and invites articles of any length. Shortening the manuscript would not allow for discussion of other treatment modalities as requested in earlier comments. We have revised the manuscript to remove unnecessary detail but maintain that this review draws attention to the lack of comparative data for these two therapies and provides a justification to clinicians to undertake further research in the form of clinical trials and observational studies. We align ourselves with the open science best practice of publication of negative findings, and also maintain this review provides a robust framework for a high quality systematic review of this topic which can be repeated in the future when further comparative data is published.*

**Reviewer #3**

Comment 1 – Paper clear and easily understood even by the non-expert. Criteria for literature selection are thoroughly described. The conclusions are interlocutory, but this is due to lack of literature. I think it can be published.

*We thank the reviewer for their kind comments.*
